# Efficacy of functional magnetic resonance imaging-guided personalized repetitive transcranial magnetic stimulation (fMRI-rTMS) in depressive patients with emotional blunting: study protocol for a randomized controlled trial

**DOI:** 10.1186/s13063-024-07976-3

**Published:** 2024-02-21

**Authors:** Yuyu Zhang, Nailong Tang, Lei Lei, Runxin Lv, Yaochi Zhang, Nian Liu, Haixia Chen, Min Cai, Huaning Wang

**Affiliations:** 1 Department of Psychiatry, Xijing Hospital, Fourth Military Medical University, Xi’an, China; 2 Department of Psychiatry, the 907th Hospital of the PLA Joint Logistics Support Force, Nanping, Fujian China

**Keywords:** Emotional blunting, FMRI-rTMS, Depression, Randomized controlled trial

## Abstract

**Background:**

Emotional blunting is a symptom that has always been present in depressed patients. Repetitive transcranial magnetic stimulation (rTMS) is a safe and effective supplementary therapy for treating depression. However, the effectiveness and brain imaging processes of functional magnetic resonance imaging-guided personalized rTMS (fMRI-rTMS) in the treatment of depression with emotional blunting have not been observed in randomized controlled trials.

**Methods:**

This study is a randomized, controlled, double-blind, and single-center clinical trial in which 80 eligible depressed patients with emotional blunting will be randomly assigned to two groups: a functional magnetic resonance imaging-guided personalized rTMS (fMRI-rTMS) group and a control group. Individuals in the fMRI-rTMS group (*n* = 40) will receive high-frequency rTMS (10 Hz, 120% MT). The main target of stimulation will be the area most relevant to the functional connectivity of the right medial prefrontal cortex (mPFC) and amygdala. The control group (*n* = 40) will receive sham stimulation, with a coil flipped to 90 degrees relative to the vertical scalp. All patients will receive 15 consecutive days of treatment, with each session lasting half an hour per day, followed by 8 weeks of follow-up. The primary outcome is the comparison of Oxford Depression Questionnaire (ODQ) scores between these two groups at different time points. The secondary outcomes include evaluating other clinical scales and assessing the differences in brain imaging changes between the two groups before and after treatment.

**Discussion:**

This trial aims to examine the effects of functional magnetic resonance imaging-guided personalized rTMS (fMRI-rTMS) intervention on depressed patients experiencing emotional blunting and to elucidate the potential mechanism behind it. The results will provide new evidence for using fMRI-rTMS in treating depression with emotional blunting in the future.

**Trial registration:**

ClinicalTrials.gov INCT05555940. Registered on 13 September 2022 at http://clinicaltrials.gov.

**Supplementary Information:**

The online version contains supplementary material available at 10.1186/s13063-024-07976-3.

## Background

Depression is characterized by feelings of sadness, lack of interest and pleasure, and decreased energy, which can seriously influence personal, work, and family relationships [1]. Antidepressants remain the preferred treatment option at present. However, mainstream antidepressants, including elective selective serotonin reuptake inhibitors (SSRIs), and serotonin-norepinephrine reuptake inhibitors (SNRIs) are often reported to cause side effects such as emotional blunting [2].

Emotional blunting manifests as a decrease in the experience of both positive and negative emotions, a lack of empathy, indifference to others, avoidance, and disregard for responsibility, which seriously affects the quality of life in depression [3]. According to Ma et al., approximately 40% to 60% of patients with depression who use SSRIs or SNRIs experience emotional blunting to varying degrees [4]. Another study also indicated that emotional blunting was not only a side effect of antidepressants but also a residual symptom of incomplete treatment for depression [5]. Furthermore, emotional blunting may decrease patient compliance and result in relapse or symptom worsening [3]. Therefore, it is necessary to research and develop methods beyond drug therapy.

An effective and noninvasive method of neuromodulation is repetitive transcranial magnetic stimulation (rTMS) [6]. Although rTMS primarily focuses on specific localized areas, it has the potential to induce modifications in brain connectivity and functional alterations in various cortical regions [7]. The modulation of distal regions is achieved by stimulating proximal regions, indicating the presence of underlying regulatory mechanisms within the brain network during this process.

A meta-analysis of major depressive disorder (MDD) found reduced coupling between the medial prefrontal cortex (mPFC) and the amygdala [8]. Both the increase and decrease in emotion activate the prefrontal cortex, and the downregulation of emotion is related to more right-lateralized activity [9]. Limbic structures, such as the amygdala, are less responsive to emotional stimuli, and subjects experience emotional blunting [10]. Using rTMS to stimulate the right mPFC, researchers found that high-frequency (20 Hz) rTMS enhanced the functional connection between the right amygdala and mPFC, while low-frequency (1 Hz) rTMS weakened the functional connection [11]. Right-sided high-frequency stimulation of the dorsolateral prefrontal cortex attenuates the processing of negatively valenced emotional stimuli in the right amygdala of healthy women [12].

In clinical practice, the transcranial magnetic stimulation site is typically set 5–6 cm in front of the motor cortex. However, this method is inaccurate [13]. According to increasing amounts of evidence, personalized interventions enhance the efficacy of transcranial magnetic stimulation therapy [14–16]. Therefore, we hypothesized that compared with the sham stimulation group, stimulating the most relevant functional connection between the right mPFC and amygdala with fMRI-rTMS could increase prefrontal cortex activity and decrease amygdala activity, thereby alleviating symptoms of emotional blunting in patients with depression.

## Methods/design

### Objective

1. The primary objective is to explore the efficacy and safety of fMRI-rTMS precise stimulation of right mPFC-amygdala individualized targets in the treatment of depression patients with emotional blunting.
2. The secondary objective is to analyze the neuroimaging mechanism that may be related to depressive emotional blunting symptoms by comparing the differences in imaging changes before and after fMRI-rTMS treatment in combination with clinical efficacy and to verify the mechanism of mPFC-amygdala functional connection in depressive emotional blunting symptoms.

### Trial design

This trial is a randomized, controlled, double-blind, parallel-group, superiority, and single-center study in which eligible participants were randomly assigned to one of two groups: (1) fMRI-rTMS or (2) sham stimulation. Both groups received a combination of antidepressant medications. The study aims to compare the improvement of emotional blunting and depressive symptoms.

**Fig. 1:**
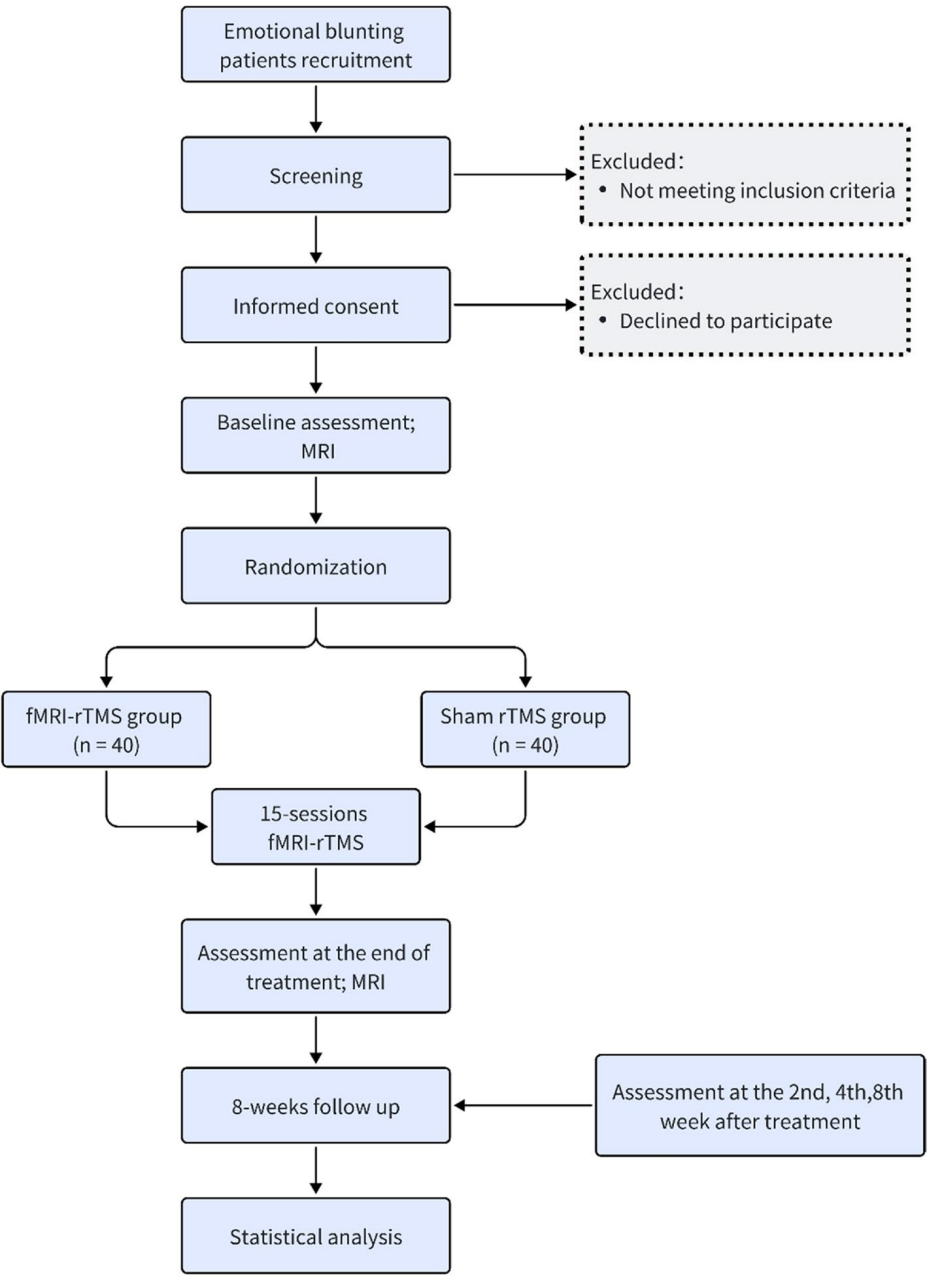
Flow chart of the trail design. *rTMS*, repetitive transcranial magnetic stimulation; *fMRI-rTMS*, functional magnetic resonance imaging-guided personalized rTMS

The Oxford Depression Questionnaire (ODQ) scores compared between the two groups at various time points served as the primary outcome measure [5]. Other depressive symptoms, such as the Montgomery and Asberg Rating Scale (MADRS) scale following the intervention, will be examined as the secondary outcome [17]. The evaluation time will be at the treatment baseline, after the 7th day of treatment, after the 15th day of treatment, and at the end of the 2nd, 4th, and 8th weekends after the treatment to further clarify the onset time and maintenance of fMRI-rTMS. In addition, structural and functional magnetic resonance imaging (MRI) is performed on the subjects using magnetic resonance imaging, combined with the precise targeted therapeutic effect of fMRI-rTMS on the functional connection of the mPFC-amygdala, to reverse verify the role of the mPFC and amygdala in the neural mechanism of depressive emotional blunting. This trial will follow the SPIRIT guidelines [18], and the SPIRIT checklist is shown in Additional file 1. The detailed flow chart is shown in Fig. 1. This study has been approved by the Ethical Committee of Xijing Hospital, Xian, China (XJLL-KY20222175) and is registered with the ClinicalTrials.gov database (NCT05555940).

### Sample size

By reviewing the literature and using the primary outcome ODQ scores, the pre-set ODQ score of the fMRI-rTMS group was 63 ± 23 [19], while the ODQ score in the sham stimulation group was 80 ± 18 [20]. The significance level for the test was set at *α* = 0.05, the power at *β* = 0.1, with a confidence level of 0.9, and the sample size was determined to be 66 cases using PASS 11 software. Because of the potential loss of experimental data or subject withdrawal from the trial, the sample size was increased by 20%. To ensure the balance of the two groups, 80 cases were finally included in the trial.

### Participants and recruitment

This trial is expected to recruit 80 depression patients with emotional blunting aged between 18 and 65. All eligible participants attend the outpatient clinic at Xijing Hospital. Before enrolling all patients in the study, we will provide a comprehensive explanation of the study’s purpose, process, associated risks, and benefits. The patients will sign the informed consent form after they understand it. Patients participating in this study were completely voluntary and had the right to withdraw at any time. Professionally trained and experienced psychiatrists will screen patients who meet the inclusion criteria and undergo clinical assessment during follow-up. To recruit more patients, we placed a recruitment advertisement in the Xijing Hospital.

### Consent or assent: ancillary studies

This trial does not involve collecting biological specimens for storage.

#### Inclusion criteria

1. Outpatients of all genders, aged ≥ 18 years and ≤ 65 years, right-handed, admitted to the psychosomatic Department of Xijing Hospital;
2. By the diagnostic criteria for depressive disorder of the Diagnostic and Statistical Manual of Mental Disorders, Fifth Edition (DSM-V);
3. Montgomery and Asberg Depression Rating Scale (MADRS): total MADRS score ≥ 12;
4. The total score of the ODQ at baseline was ≥ 50, and the answer to the standardized screening question of emotional blunting was “yes”: ‘*During the last week, to what extent have you been experiencing emotional side-effects of your antidepressant?’ The question’s subscript reads* ‘*Emotional side-effects are varied, but might include, for example — feeling emotionally ‘numbed’ or ‘blunted’ in some way/lacking positive emotions or negative emotions/feeling detached from the world around you/‘just not caring about things that you used to care about*’ [19].
5. Received at least 6 weeks of monotherapy with an SSRI or SNRI before enrollment; and
6. Subjects who can understand and are willing to strictly follow the clinical trial protocol to complete this study and sign informed consent.

#### Exclusion criteria

1. Having a history of substance abuse within 6 months before the start of the study;
2. Patients with bipolar disorder and depression caused by other mental diseases (such as psychoactive substances and non-dependent substances);
3. Having a history of severe somatic diseases or diseases that may affect the central nervous system (such as tumors and syphilis);
4. Having neurological diseases or risk of seizures, such as previous brain diseases, head trauma, alcoholism, EEG abnormalities, MRI evidence of abnormal brain structure, or family history of epilepsy;
5. There are contraindications to MRI scanning or transcranial magnetic stimulation treatment, such as metal or electronic instruments (intracranial metal foreign bodies, cochlear implants, cardiac pacemakers, stents, and other metal foreign bodies);
6. Obvious suicide risk or actual suicide behavior within 6 months before the start of the study;
7. Pregnant, breastfeeding, or planning pregnancy during the trial; and
8. Other conditions that are not suitable for the study object in the researcher’s judgment.

#### Withdrawal or dropout criteria

The investigator has the right to terminate the participant's continued participation in this study if the following conditions are met:

1. Subjects are found not to conform to the test protocol;
2. Patients who do not wish to remain enrolled in the treatment;
3. Request to change to another treatment;
4. Inability to tolerate adverse effects; and
5. Diagnosed with other psychiatric disorders after enrollment.

### Participant timeline

**Fig. 2:**
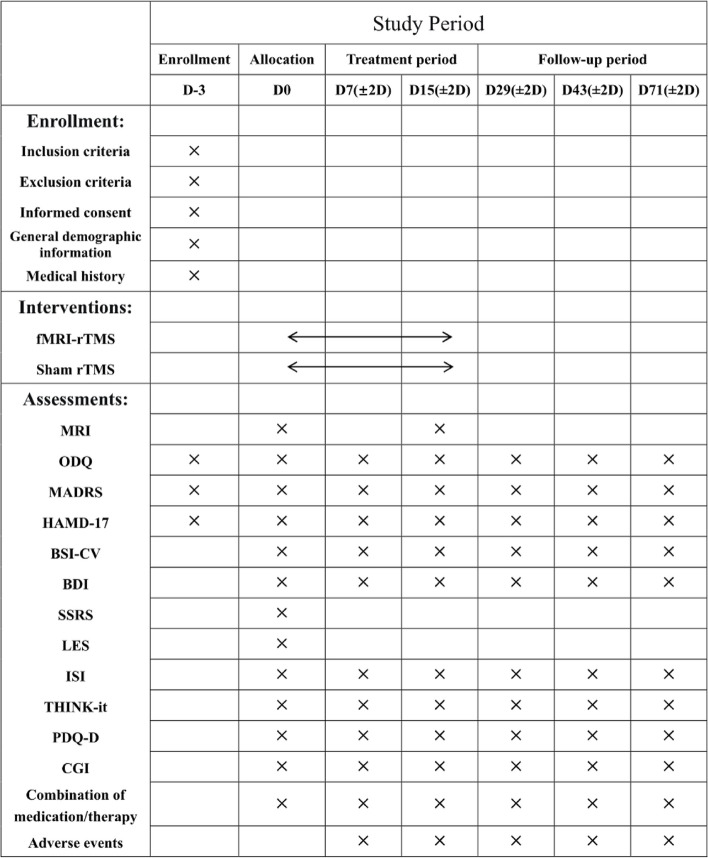
*rTMS*, repetitive transcranial magnetic stimulation; *fMRI-rTMS*, functional magnetic resonance imaging-guided personalized rTMS; *MRI*, magnetic resonance imaging; *ODQ*, Oxford Depression Questionnaire; *MADRS*, Montgomery-Asberg Depression Rating Scale; *HAMD-17*, Hamilton Depression Scale-17; *BSI-CV*, Beck Scale for Suicide Ideation-Chinese Version; *BDI*, Beck Depression Inventory; *SSRS*, Social Support Rating Scale; *LES*, Life Event Scale; *ISI*, Insomnia Severity Index; *THINK-it*, Cognitive function test; *PDQ-D*, Perceived Deficit Questionnaire for Depression; *CGI*, Clinical Global Impressions

The time schedule of enrolment, interventions, assessments, and visits for participants is shown in Fig. 2.

## Methods: assignment of interventions

### Methods: assignment of interventions

Patients who meet the inclusion criteria will be randomly assigned to two groups: the fMRI-rTMS group and the control group at a 1:1 ratio. This study will utilize Excel software to generate random numbers using the function “ = RANDBETWEEN (1,500)”. The first number will be randomly determined, and then 80 numbers will be selected to match. The block randomization method will be used to assign participants to groups. The study population that meets the inclusion criteria, which includes depression patients with emotional blunting (*n* = 80), will be randomly divided into two groups, with 40 patients in each group.

### Concealment mechanism

To ensure the concealment of the assignment process, unblinded researchers will sequentially assign participants to groups and place the grouping information in an opaque, sealed envelope. Once the study has recruited participants and collected baseline information, the unblinded researcher will provide the envelope containing the group assignment results to the treatment administrator.

### Implementation

Upon receiving a sealed envelope from an unblinded person, the treatment operator will intervene with the participant according to the grouping specified in the envelope.

### Blinding

The specific allocation and treatment plan will be kept confidential from trial participants, outcome assessors, and data analysts. The researchers who met the entry criteria and conducted a follow-up clinical evaluation are professionally trained and experienced psychiatrists. Until the conclusion of the study, neither the participants nor the researchers were aware of the categorization. Unblinding is only done in the case of patient emergencies.

### Intervention

During the study period, the subjects maintained their original antidepressant type without any changes, and the dosage remained the same as before enrollment. For patients with severe insomnia, zolpidem tartrate tablets or zopiclone tablets can be taken before bedtime. The dose should not exceed the upper limit (maximum recommended dose) specified in the instructions. During the trial, participants will be allowed to use drugs for the treatment of somatic diseases in combination, and the type and dose of drugs will remain unchanged throughout the study.

### Active fMRI-rTMS group

The Black Dolphin Transcranial Magnetic Stimulation Robot (Spirit Dolphin, SLD-YXRJ-V1.0) is the medical device used for treatment, from Xi’an Solitai Brain Control Medical Technology Co. The stimulation site of transcranial magnetic stimulation is to precisely stimulate the right mPFC-amygdala functional junction. The stimulation parameters are a frequency of 10 Hz and an MT value of 120%. Each time, 50 treatment sequences were administered, consisting of 60 stimulations per sequence, with 30-s intervals between sequences, resulting in a total of 3000 stimulations per treatment. The treatment was administered once a day for 15 days.

### Sham fMRI-rTMS group

Rotate the coil so that it is 90° away from the scalp using the widely used sham stimulation technique. The appearance of the coil, stimulation frequency, stimulation time, and duration of the stimulation period are identical to those of the actual stimulation coil. Ineffective stimulation will result because the coil’s magnetic field cannot pass through the skull to produce current. The frequency of the treatments is the same as in the control group.

### Imaging acquisition

MRI scans were performed using a 3.0 T-UNITED Discovery 770 scanner equipped with a 32-channel head coil. Earplugs are used to reduce scanner noise. They are tight but comfortable enough to minimize head motion. T1-weighted sagittal anatomical images are obtained. The parameters were as follows: resolution = 1 mm × 1 mm; repetition time (TR) = 6.0 ms; echo time (TE) = 2.4 ms; slice thickness/gap = 1 mm; acquisition matrix = 256 × 256; magnetic field strength = 3 T; flip angle = 9°; and field of view (FOV) = 256 × 256 mm. Resting-state fMRI data are as follows: resolution = 3.5 mm × 3.5 mm; TR = 2000 ms; TE = 30.0 ms; slice thickness/gap = 4.0 mm; acquisition matrix = 64 × 64; magnetic field strength = 3 T; flip angle = 90°; FOV = 224 mm × 224 mm. During the scan, all participants are instructed to keep their eyes closed, relax, and refrain from thinking about anything in particular, while ensuring they do not fall asleep.

### Follow-up

Researchers will check in with the patients by administering clinical scales and evaluating their cognitive function assessment at the end of the 2nd, 4th, and 8th weeks after treatment. The researcher will contact the patient in advance via telephone to remind them of the significance of the follow-up evaluation to ensure that the visit is not missed. Follow-up depends on the situation. Try to come to the hospital for follow-up after negotiation with the subjects, or use the self-assessment scale for telephone follow-up.

### Ancillary and post trial care

In the event of damages related to this research, compensation or indemnification will be provided by Xijing Hospital following relevant national laws and regulations.

## Outcome measures

### Primary outcome measure

Our primary outcome will be the changes in ODQ scores [5]. It is a relatively new emotional blunting assessment tool for the self-rating scale, which consists of three parts and 26 questions, focusing on the patient's emotional experience over the previous week. The first part comprised 12 questions aimed at evaluating the subjects' current experience of emotional blunting. The second part consists of 8 questions designed to compare the patient's current state with their normal state before experiencing depression. The third part included 6 questions to gauge the extent to which patients believed their emotional blunting was linked to antidepressants, as well as whether these issues affected their treatment adherence. Each question is graded on a 5-point scale, ranging from 1 (disagree) to 5 (agree). The score and total score of each dimension were summarized (total score range: 26–130). The higher the value of ODQ is, the greater the degree of emotional blunting. The Chinese version of the ODQ has been validated to have good reliability [21].

### Secondary outcome measure

1. Alterations in baseline scores on the seventeen-item Hamilton Depression Rating Scale, Montgomery-Asberg Depression Rating Scale, and other clinical scales will be used as secondary outcome measures both during treatment and at each follow-up point. The specific evaluation times were at baseline, at the end of treatment on day 7, at the end of treatment on day 15, 2 weeks after treatment, four weeks after treatment, and eight weeks after treatment.
  1. Seventeen-item Hamilton Depression Rating Scale (HAMD-17). HAMD is a widely used clinical tool for assessing the severity of depression [22]. The higher the total score, the worse the symptoms. The Chinese version of the 17-item Hamilton Depression Rating Scale demonstrated excellent interrater reliability, good item-total score correlations, and satisfactory internal reliability [23].
  2. Montgomery-Asberg Depression Rating Scale (MADRS). Each item is divided into 6 levels according to the identity level, with a score of 0–6 points. The total score of patients is 0–60. The higher the score of patients, the more severe the depression. The Chinese version exhibits high reliability, validity, and internal consistency. It is more sensitive in assessing efficacy than the HAMD [24].
  3. The Chinese Version of the Beck Scale for Suicide Ideation (BSI-CV). It is primarily used to identify individuals with suicidal tendencies and to assess the severity of suicidal thoughts. The assessment consists of 19 items, with each item offering 3 scoring options (0–2 points, respectively), resulting in a total score range of 0–38 points. A higher score indicates an elevated risk of suicide. The Chinese version exhibited high internal reliability and strong item-total correlations [25].
  4. Beck Depression Inventory (BDI). The self-rating scale is used to assess the severity of depressive symptoms. The assessment consists of 21 items, each divided into 4 levels of scores ranging from 0 to 3. The total score of the scale is the sum of the scores from all 21 entries. A higher total score indicates more severe symptoms of depression. The Chinese version demonstrates good reliability and validity in patients with depression [26].
  5. Insomnia Severity Index (ISI). The Chinese version of the ISI questionnaire has good reliability and validity in evaluating the perceived severity of insomnia [27]. The scale consists of 7 items, each scored from 0 to 3, resulting in a total score ranging from 0 to 28, with higher scores indicating poorer sleep quality.
  6. Clinical Global Impression (CGI). During each visit, the clinician evaluates the patient's overall condition, considering the severity of the illness, the patient's distress level, any other impairments, and the impact of the illness on functioning [28].
  7. Perceived Deficit Questionnaire for Depression (PDQ-D). The PDQ-D provides a subjective assessment of cognitive dysfunction. The Chinese version of the PDQ-D has been determined to be psychometrically valid for assessing subjective cognitive dysfunction in patients with depression [29]. The questionnaire comprises 20 questions, each scored from 0 to 4 points, with a total score ranging from 0 to 80 points. The higher the score, the more severe the cognitive impairment.
2. The frequency and seriousness of adverse reactions, the occurrence of adverse events connected to the treatment, and the frequency of dropouts as a result of adverse reactions.
3. Differences in brain imaging before and after therapy. Each patient underwent an MRI before and after treatment to determine the blood oxygen levels in each brain region. From this information, the functional connectivity between brain regions can be statistically determined, and the differences between the functional connectivity before and after treatment are then compared.

### Data collection plan

Baseline, assessment, and collection results will be documented on a paper Case Report Form (CRF). Data collection will be conducted by trained researchers who will receive instructions on how to perform the assessments. The two MRI scans before and after treatment will be conducted at the Yunying Medical Imaging Diagnosis Center in Xi’an, China.

### Data management

The data collected will be kept strictly confidential. Paper-based behavioral data will be periodically entered into the computer and converted into electronic data. Magnetic resonance data will also be promptly stored in the departmental database. Principal Investigators have access to the full dataset. Regular Review of Accumulated Data by the Ethics Committee of Xijing Hospital.

### Data access

Any data required to support the protocol can be supplied on request.

### Dissemination policy: reproducible research

Upon reasonable request, corresponding authors may provide the datasets used and analyzed in the current study, with the exception that participants' personal information will not be disclosed.

### Oversight and monitoring

#### Composition of the coordinating center and trial steering committee

The coordinating center includes YYZ, YCZ, NLT, LL, RXL, NL, and HXC and will be responsible for enrollment, data collection, intervention, and statistical analyses. YYZ and YCZ take charge of the implementation of the research intervention, mainly recruiting and arranging follow-up participants. RXL and NL are responsible for the data entry. The Trial Steering Committee, consisting of HNW and MC, is primarily responsible for designing and supervising the trial. The research administrators and the trial Steering Committee will convene every 2 weeks during the trial to review the trial’s progress and address implementation-related issues.

### Data monitoring: formal committee

To ensure the blinding, safety, and validity of the study, a Data Monitoring Committee (DMC) was established at the hospital. The committee consists of independent ethical, statistical, and clinical physicians and is independent of sponsors and competing interests. Any adverse reactions will be promptly reported to the DMC and relevant regulatory agencies.

### Harms

Common adverse reactions include dizziness, headache, and tingling of the scalp. The main potential adverse reactions include seizures. As a result, all researchers involved in the study will receive training related to seizures before enrolling participants to ensure their safety. Any adverse reactions will be recorded in a paper CRF and reported immediately to the DMC and relevant regulatory agencies.

### Auditing

The Ethics Committee will provide continuous regulatory oversight. The Ethics Committee will convene annually to ensure that researchers adhere to the study protocol, procedures, and regulations and that the research data obtained are objective, truthful, and integral.

### Statistical analysis

All data were analyzed based on the intention-to-treat principle, aiming to maintain the true state of the intervention as accurately as possible. Multiple imputations will be used to handle missing data.

#### Data analysis

1. Data analysis before intervention

The chi-square or Kruskal–Wallis tests will be used to examine all sociodemographic information, as well as the results of the pretreatment scale. Baseline information between groups will be analyzed using either a paired t-test or a rank sum test. All tests are two-sided tests, with a significance level of *p* ≤ 0.05.

2. Overall clinical scale data analysis

The ODQ scale, MADRS scale, Beck Scale for Suicide Ideation-Chinese Version (BSI-CV), and other scores will be examined during the treatment and follow-up period using repeated measures analysis of variance to examine differences between groups before and after the intervention.

#### MRI data analysis

SPM and other tools will be used to statistically evaluate the magnetic resonance data. The differences between the experimental and control groups as well as changes in imaging indicators such as functional connectivity, low-frequency amplitude, and local consistency will be investigated using the double-sample *t*-test.

### Interim analyses

There will be no interim analysis. There are no expected issues that would harm the participant.

### Dissemination policy: trial results

The results of this study will be published in an international peer-reviewed journal.

## Discussion

Previous research has shown that switching to vortioxetine can considerably reduce emotional blunting in depressed patients who were taking SSRIs or SNRIs [19].

Nevertheless, to our knowledge, neuromodulation therapies have not been used to treat emotional blunting in patients with depression. An increasing number of studies have demonstrated that fMRI-rTMS may be a safe and effective treatment for depression. It is still unknown, however, whether these advantages also apply to the symptoms of emotional blunting experienced by depressed people. This study can not only observe the efficacy and safety of imaging-guided personalization of transcranial magnetic stimulation on the right side of the connection between the mPFC and amygdala but also explore the neuroimaging mechanism related to emotional blunting in depression. Furthermore, it aims to investigate the pathophysiological issues associated with emotional blunting. This will be valuable for designing larger and more comprehensive clinical randomized trials and for locating more potent targets for the fMRI-rTMS treatment of emotional blunting.

## Trial status

The trial is still recruiting patients. The first patient was recruited into the group on February 15, 2023, and it is expected to end in June 2024. Patients for this trial are still being recruited. This is version 4.0 of the protocol, dated 10 September 2023.

### Supplementary Information

**Supplementary Material 1.**

**Supplementary Material 2.**

## Data Availability

Not applicable.

## Abbreviations

rTMS: Repetitive transcranial magnetic stimulation
fMRI-rTMS: Functional magnetic resonance imaging-guided personalized rTMS
mPFC: Medial prefrontal cortex
SSRI: Selective serotonin reuptake inhibitor
SNRI: Serotonin-norepinephrine reuptake inhibitor
ODQ: Oxford Depression Questionnaire
MADRS: Montgomery and Asberg Rating Scale MRI
DSM-V: Diagnostic and Statistical Manual of Mental Disorders, Fifth Edition
MRI: Magnetic resonance imaging
TR: Repetition time
TE: Echo time
FOV: Field of view
HAMD-17: Hamilton Rating Scale for Depression
BSI-CV: Chinese Version of Beck Scale for Suicide Ideation
BDI: Beck Depression Inventory
ISI: Insomnia Severity Index
CGI: Clinical Global Impression
PDQ-D: Perceived Deficit Questionnaire for Depression
CRF: Case Report Form
DMC: Data Monitoring Committee
