# Supplementary material for: Efficacy of functional magnetic resonance imaging-guided personalized repetitive transcranial magnetic stimulation (fMRI-rTMS) in depressive patients with emotional blunting: study protocol for a randomized controlled trial
Source: Trials. 2024 Feb 21;25:134. doi: 10.1186/s13063-024-07976-3 (PMC10880253; doi:10.1186/s13063-024-07976-3)
Supplement: Supplementary file 2 — Supplementary Material 2. [file 13063_2024_7976_MOESM2_ESM.docx]

**Efficacy and brain imaging mechanisms of individualized precise transcranial magnetic stimulation for emotional blunting in depression based on image analysis**

Applicant: Xijing Hospital

Project leader: Huaning Wang

Study period: January 30, 2022 - June 30, 2024

Version number: V4.0

Program No.: XJLL-KY20222175

**Version date: September 10, 2023**

Dear patient,

We would like to invite you to take part in a pilot study that will examine “Efficacy and brain imaging mechanisms of individualized precise transcranial magnetic stimulation for emotional blunting in depression based on image analysis”. The study will be conducted by Associate Professor Wang Huaning. China's laws and regulations, as well as the Declaration of Helsinki, will be strictly followed during the study's execution.

Please carefully read the following information before deciding whether or not to participate in this clinical research study. It will help you understand the purpose of the study, its procedures, and duration, as well as the potential benefits, risks, and discomforts you may experience. You can talk to your friends and family about this and ask the study's lead physician any questions you may have. The physician will answer your inquiries and assist you in coming to a decision.

**I. Background of the study**

Depression is a common mental illness that is traditionally treated with medication, but many patients do not respond well to medication and suffer from side effects such as emotional blunting. Emotional blunting refers to the numbing of positive and negative emotions. It is estimated that about 40-60% of depressed patients using selective serotonin reuptake inhibitors (SSRIs), and serotonin-norepinephrine reuptake inhibitors (SNRIs) have varying degrees of dysphoria. Repetitive transcranial magnetic stimulation (rTMS) is a safe and noninvasive treatment for depression, and its clinical efficacy is related to the accuracy of stimulation targeting. How to carry out individualized and precise targeting is important to improve therapeutic efficacy. How to perform individualized and precise targeting is important for improving treatment efficacy.

**II. Purpose of the study**

This study aims to explore the brain imaging mechanisms that may be associated with the symptoms of emotional blunting in depression through magnetic resonance imaging, and then to guide rTMS for individualized and precise localization of the symptoms of emotional blunting in depression based on resting-state functional magnetic resonance through the method of functional connectivity of the deep brain regions, to explore and examine the therapeutic effect and mechanism of action of the new individualized localized stimulation therapy on the symptoms of emotional blunting, to provide a new therapeutic means and a more desirable therapeutic efficacy for this disease.

**III. Study design**

The Psychosomatic Department of the First Affiliated Hospital of the Air Force Medical University conducted this study. In this study, eighty patients just like you are taking part.

When you understand the possible benefits and risks of participating in the trial, voluntarily participate and sign the informed consent approved by the Ethics Committee, and when the clinician judges that you may be eligible for enrollment, relevant information will be collected, and relevant examinations and assessments will be performed on you, including demographic information; detailed information on whether you have ever suffered from any diseases, your family history, and previous suicides, etc.; completion of various clinical and cognitive scales scores We will perform a cranial magnetic resonance (MRI) examination, collect facial MRI data, perform 3D facial modeling, create individualized 3D printed facial tracers, and identify treatment targets for precision navigation. If you are eligible for enrollment and receive rTMS precision-targeted therapy, you have a 50% chance of being assigned to the sham group. The study was divided into a treatment period of 15 days, with each treatment session lasting approximately 30 minutes, once a day, and a follow-up period of 8 weeks, with clinical scales and cognitive function assessments at baseline, at the end of treatment on day 7, at the end of treatment on day 15, at the end of treatment on weekends 2, 4, and 8, respectively. Magnetic resonance scans were performed at baseline and the end of treatment on day 15 in all groups. Follow-up visits were done as appropriate, in consultation with the subjects to try to come to the hospital for follow-up, otherwise, telephone follow-up was done using self-assessment scales.

**IV. Inclusion and exclusion criteria**

**This is a pilot study and to participate in the program you will need to meet the following criteria:**

(1) Outpatients of all genders, aged ≥18 years and ≤65 years, right-handed, admitted to the psychosomatic Department of Xijing Hospital;

(2) By the diagnostic criteria for depressive disorder of the Diagnostic and Statistical Manual of Mental Disorders, Fifth Edition (DSM-V);

(3) Montgomery and Asberg Depression Rating Scale (MADRS): total MADRS score ≥12;

(4) The total score of the ODQ at baseline was ≥50, and the answer to the standardized screening question of emotional blunting was "yes": ‘During the last week, to what extent have you been experiencing emotional side-effects of your antidepressant?’ The question's subscript reads ‘Emotional side-effects are varied, but might include, for example — feeling emotionally ‘numbed’ or ‘blunted’ in some way/lacking positive emotions or negative emotions/feeling detached from the world around you/ ‘just not caring about things that you used to care about.’

(5) Received at least 6 weeks of monotherapy with an SSRI or SNRI before enrollment;

(6) Subjects who can understand and are willing to strictly follow the clinical trial protocol to complete this study and sign informed consent.

**Moreover, the following conditions are not satisfied:**

(1) Having a history of substance abuse within 6 months before the start of the study;

(2) Patients with bipolar disorder and depression caused by other mental diseases (such as psychoactive substances and non-dependent substances);

(3) Having a history of severe somatic diseases or diseases that may affect the central nervous system (such as tumors, syphilis, etc.);

(4) Having neurological diseases or risk of seizures, such as previous brain diseases, head trauma, alcoholism, EEG abnormalities, MRI evidence of abnormal brain structure, or family history of epilepsy;

(5) There are contraindications to MRI scanning or transcranial magnetic stimulation treatment, such as metal or electronic instruments (intracranial metal foreign bodies, cochlear implants, cardiac pacemakers, stents, and other metal foreign bodies);

(6) Obvious suicide risk or actual suicide behavior within 6 months before the start of the study;

(7) Pregnant, breastfeeding, or planning pregnancy during the trial;

(8) Other conditions that are not suitable for the study object in the researcher's judgment.

**The following situations give the researcher the authority to stop you from continuing to participate in this study:**

(1) You do not meet the inclusion criteria;

(2) For reasons of your health, the researchers believe that it is in your best interest to choose another treatment;

(3) Other risk factors are identified during the study and it is determined that you are not a good candidate for continued participation in this study;

(4) Legal and regulatory reasons;

(5) Termination of the entire study.

**V. Research process**

During the roughly ten weeks that the trial will last, you will be required to undergo pre-treatment testing, data collection, transcranial magnetic stimulation treatment, and post-treatment clinical monitoring and assessment. A 15-day treatment phase and an 8-week follow-up phase comprise the study's design. MRI scans were performed on the patients both at the beginning and end of the 15-day treatment period. Individualized 3D printed facial tracers were made in conjunction with the facial MRI data after the initial MRI scan, and subsequent rTMS would be based on this facial tracer to accurately simulate the target targets. Individualized mPFC-amygdala stimulation targets were identified based on the functional phase MRI data. Clinical scales and cognitive function assessments were performed at baseline, at the end of treatment on day 7, at the end of treatment on day 15, and at the end of treatment on weekends 2, 4, and 8, respectively, and subjects were negotiated to try to come to the hospital for follow-up, otherwise, telephone follow-up was performed using self-assessment scales.

**VI. Risks and discomforts**

Except for those who have electronic, magnetic, or mechanical devices on their bodies (e.g., pacemakers, metal dentures), or those who have intracranial artery entrapment, MRI is known to have very few hazards or side effects on the human body. The greatest risk is that metal attracted to a magnetic field will strike you during its flight motion. To minimize this risk, we ask that all participants in the exam remove all metal objects from their bodies before entering the magnet room.

MRI scans are not uncomfortable, but please let us know if you are afraid of enclosed spaces. The first scan lasts about 50 minutes and subsequent scans last about 30 minutes. There will be noise during the scan, and the first few minutes of the first scan will be relatively noisy to better help reconstruct individualized brain information. You will be given earplugs to minimize the noise.

Keeping the head still during the experiment is very important for this study. The padding used to hold the head in place generally feels comfortable, but you should tell the experimenter if you feel uncomfortable. You may stop the experiment at any time during the experiment without any reason.

The main risk and discomfort of neuropsychological testing is primarily fatigue. You may request a break at any time during the test.

Repetitive Transcranial Magnetic Stimulation (rTMS) is a non-invasive intervention that has been approved in several countries for disorders such as depression, obsessive-compulsive disorder, and neuropathic pain, and is also widely used for insomnia, addiction, Alzheimer's disease, Parkinson's disease, and post-stroke rehabilitation. Except for people with electronic, magnetic, or mechanical devices (e.g., pacemakers, metal dentures), rTMS has few harmful effects or side effects on the human body, commonly minor headaches and dizziness, which are relieved naturally without any special treatment. rTMS has a very low incidence of inducing epilepsy, with only 24 epileptic seizures occurring in all the rTMS studies reported so far, less than 1 in 10,000, which is associated with The incidence of rTMS-induced seizures extremely low, with only 24 seizures in rTMS studies reported so far, which is less than 1 in 10,000, and is closely related to the stimulation parameters (e.g., frequency, intensity, duration, intervals) and the coil type used, and even lower with the stimulation parameters and coils used in the present study. rTMS-induced seizures are self-limiting and transient and do not have any long-term effects.

If you experience any of the above discomforts or other accidents, please tell your study physician immediately, and we will deal with them carefully and promptly. In the event of a serious adverse event such as a seizure, the experiment will be stopped immediately and arrangements for hospitalization or adjustment of the treatment plan will be coordinated.

**VII. Possible benefits of participation in the study**

Direct Benefit: Your condition will likely improve during the study period, which is the direct medical benefit of your participation in this study; you will receive specialized care from your doctor, as well as close follow-up visits to guide your recovery.

Indirect Benefit: The medical data you provide by participating in this study may help benefit more patients with the same condition as yours, adding new and useful information to medicine.

**VIII. Costs of the study**

During the study period, after you sign the informed consent form, the scale assessment, neurocognitive function test, magnetic resonance examination, 3D printed mask of the navigation robot and transcranial magnetic stimulation treatment will be provided free of charge to you by the subject group. The antidepressants and other medications you take as well as the transportation costs incurred during the follow-up visits will be your responsibility.

**IX. Compensation and reparation for research**

In the event of damages related to this research, compensation or indemnification will be made by Xijing Hospital according to relevant national laws and regulations.

**X. Alternative therapies**

Participation in this study is not necessary to treat your disease or condition. There are other treatment options available for your condition, such as reducing the dose of your medication or switching to another class of antidepressant medication. The benefits and risks will be related to the specific treatment regimen, and while you may gain control or relief from your disease, you may also experience some of the toxic side effects that are common with treatment. Your study doctor will talk to you about your specific treatment plan and explain what good and bad things can happen when you receive other treatment options. He/she will answer all your questions.

**XI. Confidentiality of personal information**

The hospital will keep the information collected in this study confidential. To protect your identity, a uniform format number, rather than your name, will be used for any information about you in the study documents. Any information that could help identify you will be removed from all subject information collected and combined, ensuring that the information cannot be linked to a specific study subject.

To the extent permitted by applicable laws and regulations and without violating the privacy of the subject, the Ethics Committee and regulatory authorities will have direct access to the subject's original medical records to verify the clinical trial procedures and/or data (not available to the subject group), and you or your legal representative will authorize such access by signing the informed consent form. To the extent permitted by applicable laws and/or regulations, records identifying you will be kept confidential and will not be made public, and the results of this study may be published in medical journals shared for scientific purposes or used by the subject group for product research or improvement, but your identity and personal information will not be disclosed at any time.

**XII. Exit studies**

During the study, the investigator will take into account your best interests, and if he/she feels that you are no longer suitable to continue with the trial (including recurrence of disease, intolerable toxicity, and serious adverse events, etc.), or if the study group/ethics committee/government requests that the study be discontinued, the investigator will take the initiative in explaining the reasons and discontinuing your participation in this drug study. Your participation in this study is completely voluntary and you have the right to choose not to participate in this study and to withdraw at any time without penalty or loss of benefits, and your subsequent treatment will not be affected in any way. If you are ready to withdraw from the study, please tell your study doctor promptly, who will conduct a thorough examination and treatment for your safety. The investigator will promptly inform you or your guardian when he or she gets information that may affect your continued participation in the trial.

**XIII. Contact Person and Contact Information**

You will be kept informed of the information and progress of the study, and you will be notified of any new information about the safety of the study. If you have questions about this study, experience any discomfort or injury during the study, or have questions about the rights of participants in this study, you may contact (researcher: Yuyu Zhang) at (phone number: 15535849017).

**XIV. Contact information of the Ethics Committee**

If you have any questions or claims about your rights and health as a result of participating in this study, you may contact the Ethics Committee of Xijing Hospital at 029-84771794.

**Subject Informed Consent Signature Page**

I have read the above-informed consent form in detail and understand the purpose of this study and the possible benefits and risks of participating in this study. The researchers have explained the medical terms clearly. I have been allowed to ask questions and all questions have been answered in an easy-to-understand manner. I may choose not to participate in this study or to withdraw at any time by notifying the doctor in charge, and none of my medical treatment or rights will be affected. The Physician-in-Charge may terminate my continued participation in this study if I need other treatment if I do not comply with the study plan, if a study-related injury occurs, or for any other reason.

**I have read the above-informed consent form and have a copy of it, which my doctor has fully explained to me. I'm taking part in this clinical trial voluntarily.**

Name of participant in block letters:

Participant's signature: Date:

Phone number of participant:

Legal representative name in block letters: (if applicable)

Relationship with the participant:

Legal representative signature: Date:

Phone number of legal representative:

Name of Witness in block letters: (if applicable)

Signature of witness: Date:

| **I confirm that I have thoroughly explained to the patient about this clinical trial, including the patient's possible benefits and risks, and have answered all questions asked by the patient.** |
| --- |

Doctor's signature: Date:

Phone number of the doctor:
